# Signaling Lymphocyte Activation Molecule Family 5 Enhances Autophagy and Fine-Tunes Cytokine Response in Monocyte-Derived Dendritic Cells *via* Stabilization of Interferon Regulatory Factor 8

**DOI:** 10.3389/fimmu.2018.00062

**Published:** 2018-01-26

**Authors:** Zsofia Agod, Kitti Pazmandi, Dora Bencze, Gyorgy Vereb, Tamas Biro, Attila Szabo, Eva Rajnavolgyi, Attila Bacsi, Pablo Engel, Arpad Lanyi

**Affiliations:** ^1^Department of Immunology, Faculty of Medicine, University of Debrecen, Debrecen, Hungary; ^2^Department of Biophysics and Cell Biology, Faculty of Medicine, University of Debrecen, Debrecen, Hungary; ^3^Department of Bioengineering, Sapientia Hungarian University of Transylvania, Cluj-Napoca, Romania; ^4^Department of Biomedical Sciences, Medical School, University of Barcelona, Barcelona, Spain

**Keywords:** SLAMF5, autophagy, dendritic cell, IRF8, TRIM21, IL-12p70, LPS/IFNγ

## Abstract

Signaling lymphocyte activation molecule family (SLAMF) receptors are essential regulators of innate and adaptive immune responses. The function of SLAMF5/CD84, a family member with almost ubiquitous expression within the hematopoietic lineage is poorly defined. In this article, we provide evidence that in human monocyte-derived dendritic cells (moDCs) SLAMF5 increases autophagy, a degradative pathway, which is highly active in dendritic cells (DCs) and plays a critical role in orchestration of the immune response. While investigating the underlying mechanism, we found that SLAMF5 inhibited proteolytic degradation of interferon regulatory factor 8 (IRF8) a master regulator of the autophagy process by a mechanism dependent on the E3-ubiquitin ligase tripartite motif-containing protein 21 (TRIM21). Furthermore, we demonstrate that SLAMF5 influences the ratio of CD1a^+^ cells in differentiating DCs and partakes in the regulation of IL-1β, IL-23, and IL-12 production in LPS/IFNγ-activated moDCs in a manner that is consistent with its effect on IRF8 stability. In summary, our experiments identified SLAMF5 as a novel cell surface receptor modulator of autophagy and revealed an unexpected link between the SLAMF and IRF8 signaling pathways, both implicated in multiple human pathologies.

## Introduction

Macroautophagy (here referred to as autophagy) is a conserved catabolic pathway, whereby cytosolic contents are sequestered by *de novo* formed double-membrane-bound vesicles, called autophagosomes and carried to lysosomes for degradation. It is active at basal levels in most cell types to recycle macromolecules (1, 2) and to prevent accumulation of cytotoxic metabolites (3). Beyond maintaining cellular homeostasis, autophagy improves cell autonomous and host defense mechanisms against a number of pathogens by regulating intracellular protein trafficking and degradation as well as antigen presentation (4, 5). In addition, autophagy guards against both untimely and excessive inflammatory reactions by influencing the activation and duration of inflammation *via* suppression of ROS accumulation and removal of danger signals as well as regulation of pro-inflammatory cytokine production (6).

Dendritic cells (DCs) continuously migrate from tissues to lymph nodes to present antigens to antigen-specific T cells. The DC pool of non-lymphoid organs is maintained by constant replenishment from circulating monocytes (7, 8) whose differentiation into DCs is dependent on the induction of autophagy (9). DCs exploit autophagy to display cytoplasmic self- or foreign antigens on MHC II molecules for CD4^+^ T cells (10). This mechanism, depending on the presence or absence of danger signal-induced co-stimulation, contributes to the initiation of a pathogen-specific immune response and to establishment or maintenance of peripheral tolerance, respectively (11). The rate of autophagy therefore must be stringently controlled to adapt to the actual immune context. Stimulation of DCs by LPS has been shown to transiently reduce autophagy and its associated functions (12), presumably to diminish presentation of self-antigens and focus the immune response against an emerging environmental threat. However, as all immune responses, including TLR-mediated functions have the potential to convey damage to host tissues, the recovery of autophagy, reestablishing its anti-inflammatory effects is increasingly recognized as an essential component of the maintenance of host tissue integrity.

Recent work of the Ozato laboratory identified interferon regulatory factor 8 (IRF8) as a positive regulator of autophagy in murine macrophages and DCs exposed to various stress signals, including starvation, exposure to TLR ligands or infection with *Listeria monocytogenes* (13). Furthermore, their earlier work demonstrated that stimulation of murine macrophages with LPS/FNγ induced secretion of IL-12 that was fully dependent on IRF8 (14). The amount and activity of the IRF8 protein were found to be controlled by ubiquitin ligases (TRIM21, c-Cbl), the p62 ubiquitin-binding protein (Sequestosome-1) as well as the deubiquitinase USP4, regulating its proteasomal degradation (15–18). The tasks of IRF8 as a regulator of autophagy or its role in human monocyte-derived dendritic cells (moDCs) functions have not been properly addressed.

Members of the cell surface-expressed signaling lymphocyte activation molecule family (SLAMF) receptors (19–21) have been shown to regulate autophagy. SLAMF1 (CD150) and SLAMF4 (CD244 or 2B4) were reported to bind to the Beclin-1/Vps34 autophagy-associated complex (22–24) responsible for generation of PI(3)P, a phospholipid involved in autophagic vesicle nucleation. SLAMF1 increased the autophagic flux in human chronic lymphocytic leukemia cells (25) *via* stabilization of the above autophagic macrocomplex. On the contrary, SLAMF4 was identified as an inhibitor of starvation- and rapamycin-induced autophagy in human lymphoblastoid cell lines and in murine bone marrow-derived macrophages *via* reducing Vps34 lipid kinase activity (23).

SLAMF5 is a self-ligand receptor broadly expressed on the surface of hematopoietic cells that during cell–cell communication acts both as an adhesion and signaling molecule (26–28). Although its cell surface expression on both the myeloid and plasmacytoid subsets of DCs have been established (29, 30), its function in these cells has not been addressed.

Overall, regulatory circuits of autophagy and inflammation are interconnected at multiple levels (4–6), thus molecules involved in the regulation of autophagy have a major impact on the outcome of the immune response. Identification of autophagy regulators, cell surface receptors, readily accessible for antibodies in particular, may provide excellent targets to modulate autophagy in various disease states.

In this report, we examined the effect of SLAMF5 on human moDC responses induced by LPS/IFNγ, frequently used as a model of infection with Gram-negative bacteria. We identify SLAMF5 as a novel cell surface-expressed regulator of basal autophagy that responding to inflammatory signals fine-tunes cytokine production of human moDCs *via* affecting a pathway responsible for proteasomal degradation of IRF8.

## Materials and Methods

### *In Vitro* Differentiation of moDC and RNA Interference

Monocytes were isolated from human leukocyte concentrates (buffy coats) obtained from healthy blood donors drawn at the Regional Blood Center of Hungarian National Blood Transfusion Service (Debrecen, Hungary) in accordance with the written approval of the Director of the National Blood Transfusion Service and the Regional and Institutional Ethics Committee of the University of Debrecen, Faculty of Medicine (Debrecen, Hungary). Peripheral blood mononuclear cells (PBMC) were separated by Ficoll Paque (GE Healthcare) gradient centrifugation, and then monocytes were purified by magnetic cell isolation using CD14 antibody-coated magnetic microbeads (Miltenyi Biotech) according to the manufacturer’s protocol. The following siRNAs were used for gene silencing by transfection of freshly isolated primary human monocytes:

SLAMF5 sense: 5′-UGGCUAUGUUCUUUCUGCUUGUUCU-3′,
SLAMF5 antisense: 5′-AGAACAAGCAGAAAGAACAUAGCCA-3′,
Negative control for SLAMF5 sense: 5′-UGGUAUGCUUUCUGUUCGUUUCUCU-3′,
Negative control for SLAMF5 antisense: 5′-AGAGAAACGAACAGAAAGCAUACCA-3′.

(Stealth™ RNAi, ThermoFisher Scientific), IRF8 Silencer Select siRNA (Assay ID: s7100), TRIM21 Silencer Select siRNA (Assay ID: s13462), and non-targeted Silencer Select Negative Control No 1. siRNA (ThermoFisher Scientific). The siRNA duplexes were delivered by electroporation using GenePulser Xcell instrument (Bio-Rad Laboratories). Following transfection, monocytes were cultured at a density of 10^6^ cells ml^−1^ on 24-well plates in complete RPMI-1640 medium containing 10% fetal bovine serum (both from ThermoFisher Scientific), 100 U ml^−1^ penicillin and 100 ng ml^−1^ streptomycin (both from Sigma-Aldrich), supplemented with 80 ng ml^−1^ GM-CSF (Gentaur Molecular Products), and 100 ng ml^−1^ IL-4 (PeproTech) for 5 days to generate moDCs. Culture medium was replenished on day 2 by removing three-quarters of the supernatant and replacing it by complete medium containing GM-CSF and IL-4.

### Receptor Cross-linking

For SLAMF5 stimulation moDCs adjusted to 10^7^ ml^−1^ were suspended in complete RPMI-1640 medium containing 10 µg ml^−1^ anti-SLAMF5 antibody (clone 152-1D5; LifeSpan BioSciences, Cat. No. LS-C134663) or an IgG isotype control antibody (BioLegend, Cat. No. 400124) at 4°C for 45 min. Thereafter, following thorough washing procedures, cells were reseeded into 24-well cell culture plates and incubated in complete medium in the presence of 10 µg ml^−1^ F(ab′)_2_ of goat anti-mouse IgG (Jackson ImmunoResearch Laboratories, Cat. No. 115-006-062) at 37°C for 2 h.

### Cell Stimulation

Dendritic cell maturation was induced by simultaneous addition of 100 ng ml^−1^ LPS (ultrapure lipopolysaccharide from *Salmonella minnesota* R595, Cat. No. tlrl-smlps) and 10 ng ml^−1^ recombinant human IFNγ (PeproTech, Cat. No. 300-02) for the indicated time periods. For autophagy induction, moDCs were exposed to 50 nM rapamycin (Merck, Cat. No. 553210) for 4 h. The optimal concentration of rapamycin was determined as one that readily increased LC3-II levels without significant toxicity. In some experiments, cells were incubated with 20 nM bafilomycin A1 (BafA1) (InvivoGen, Cat. No. tlrl-baf1) or 1 µM MG132 (SelleckChem, Cat. No. S2619) for the last 2 h.

### Western Blot Analysis

For western blot analysis protein extracts were obtained by lysing cells in Laemmli buffer. The samples were resolved by SDS-PAGE and transferred electrophoretically onto nitrocellulose membranes (Bio-Rad Laboratories). Non-specific binding was blocked by TBS–Tween–5% non-fat dry milk, and then the membrane was probed with anti-SLAMF5 (clone H128), anti-β-actin (Cat. No. sc-47778), anti-Akt1 (Cat. No. sc-5298), anti-ubiquitin (Cat. No. sc-9133), and anti-TRIM21 (Cat. No. sc-25351) all from Santa Cruz Biotechnology, anti-phospho-p70S6K Thr389 (Cat. No. 9206), anti-p70S6K (Cat. No. 9202), and anti-IRF8 (Cat. No. 5628S) all from Cell Signaling, anti-phospho-Akt Ser473 (R&D Systems, Cat. No. AF887), anti-EAT-2 (LifeSpan BioSciences, Cat. No. LS-C169054) or anti-LC3 (Novus Biologicals, Cat. No. NB100-2220) antibodies followed by washing and incubation with anti-mouse or anti-rabbit horseradish peroxidase-conjugated secondary antibodies (GE Healthcare). Specific signals were detected on X-ray films using the ECL system (SuperSignal West Pico/Femto chemiluminescent substrate; ThermoFisher Scientific). Protein bands were scanned, and band densities were determined using Kodak 1D Image Analysis software, version 3.6. Relative density was calculated by normalizing to β-actin band intensities. The level of phosphorylation was normalized to the total amount of the same protein present in the samples.

### RNA Extraction, Reverse Transcription and Real-time Quantitative PCR

Extraction of total RNA was performed using TRI-Reagent (Molecular Research Center) according to the protocol of the manufacturer. Total RNA was treated with DNase I (ThermoFisher Scientific), and then cDNA was synthesized using the High Capacity cDNA Reverse Transcription kit of Applied Biosystems. Quantitative PCR was performed with the ABI StepOne Real-Time PCR System using Dream Taq DNA Polymerase (ThermoFisher Scientific) and gene-specific assay for IRF8 (ThermoFisher Scientific, Assay ID: Hs00175238_m1) according to the manufacturer’s instructions. The cycle threshold (Ct) values were determined using the StepOne v2.1 Software (Applied Biosystems). The relative amount of mRNA (2^−ΔCt^) was obtained by normalizing to the cyclophilin housekeeping gene in each experiment.

### Flow Cytometry

Viability of the cells was determined on day 5 by 7-aminoactinomycin-D (7-AAD 10 µg ml^−1^; Sigma-Aldrich) staining for 15 min immediately before flow cytometry. Cell surface protein expression was detected with FITC-labeled monoclonal antibodies against HLA-A, B, C (Sony Biotechnology), HLA-DQ, HLA-DR, and CD40 (all from BioLegend), PE-labeled monoclonal antibodies against SLAMF5 (BioLegend, clone 1.21), CD14 and CD86 (both from R&D Systems), DC-SIGN (Sony Biotechnology), and APC-labeled monoclonal antibodies against CD1a (BioLegend). Isotype-matched control antibodies were obtained from BioLegend. Measurement of autophagy was performed using the CYTO-ID Autophagy detection kit (Enzo Life Sciences) according to the manufacturer’s instructions. Briefly, 2 × 10^5^ cells were incubated with CYTO-ID Green autophagy detection dye (1:2,000) for 30 min at 37°C, then cells were washed and immediately subjected to flow cytometry. Fluorescence intensities were measured with a FACS Calibur cytometer (BD Biosciences) excluding cell debris by forward/side scatter gating. In case of CD1a analysis, the positive gate was set excluding the area staining with the isotype-matched control mAb. Relative fluorescence and the percentage of positive cells in samples were determined with the FlowJo software.

### Confocal Microscopy

For live cell confocal microscopy, 2 × 10^5^ cells were cultured on 8-well μ-Slides (chambered coverslip, ibidi GmbH). Where indicated, 50 µM Chloroquine (Enzo Life Sciences) was used to inhibit autophagosome turnover. Cells were stained with CYTO-ID Green Detection Reagent (1:2,000) and Hoechst 33342 Nuclear Stain (1:2,000) for 30 min at 37°C. Next, cells were washed as recommended by the manufacturer and visualized immediately by a Zeiss LSM880 confocal microscope equipped with an NA = 1.2, 40× water immersion objective. Hoechst was excited at 405 nm and detected in the 410–485 nm range, while CYTO-ID was excited at 488 nm and detected in the 499–562 nm range. Scanning was done at a 0.86 μs/pixel rate in multitrack/line switch mode to avoid spectral spillover. Pinhole was set to 32 µm, equivalent to 1 Airy unit. Lateral (*x*–*y*) sampling was 0.87 μm/pixel. Images were obtained within 45 min after staining to avoid loss of fluorescence due to leakage of the dye.

### ELISA

The concentrations of IL-1β and IL-12 in culture supernatants were determined by BD-OptEIA Human ELISA kits (BD Biosciences), and the level of IL-23 was measured with the human IL-23 ELISA Ready-Set Go kit (eBioscience) according to the manufacturer’s instructions. Absorbance measurements were carried out by a Synergy HT microplate reader at 450 nm.

### Statistical Analysis

We performed parallel experiments and compared matching observations on control and silenced cells, thus the research design meets the assumptions of the paired *t*-test. However, following monocyte separation, we randomly assigned cells from the same donor to either the knockdown or control groups, cultured the transfected cells separately for 5 days, and then acquired post-intervention data from the two populations. For these reasons, we considered our samples independent and determined the statistical differences between the experimental groups by unpaired two-tailed Student’s *t*-test. *p*-values of <0.05 were considered to be statistically significant. All data were derived from at least three independent experiments and are expressed as mean ± SD. Data were analyzed with the Prism software (GraphPad).

## Results

### SLAMF5 Is Upregulated during Differentiation and Activation of Human moDCs

**Figure 1:**
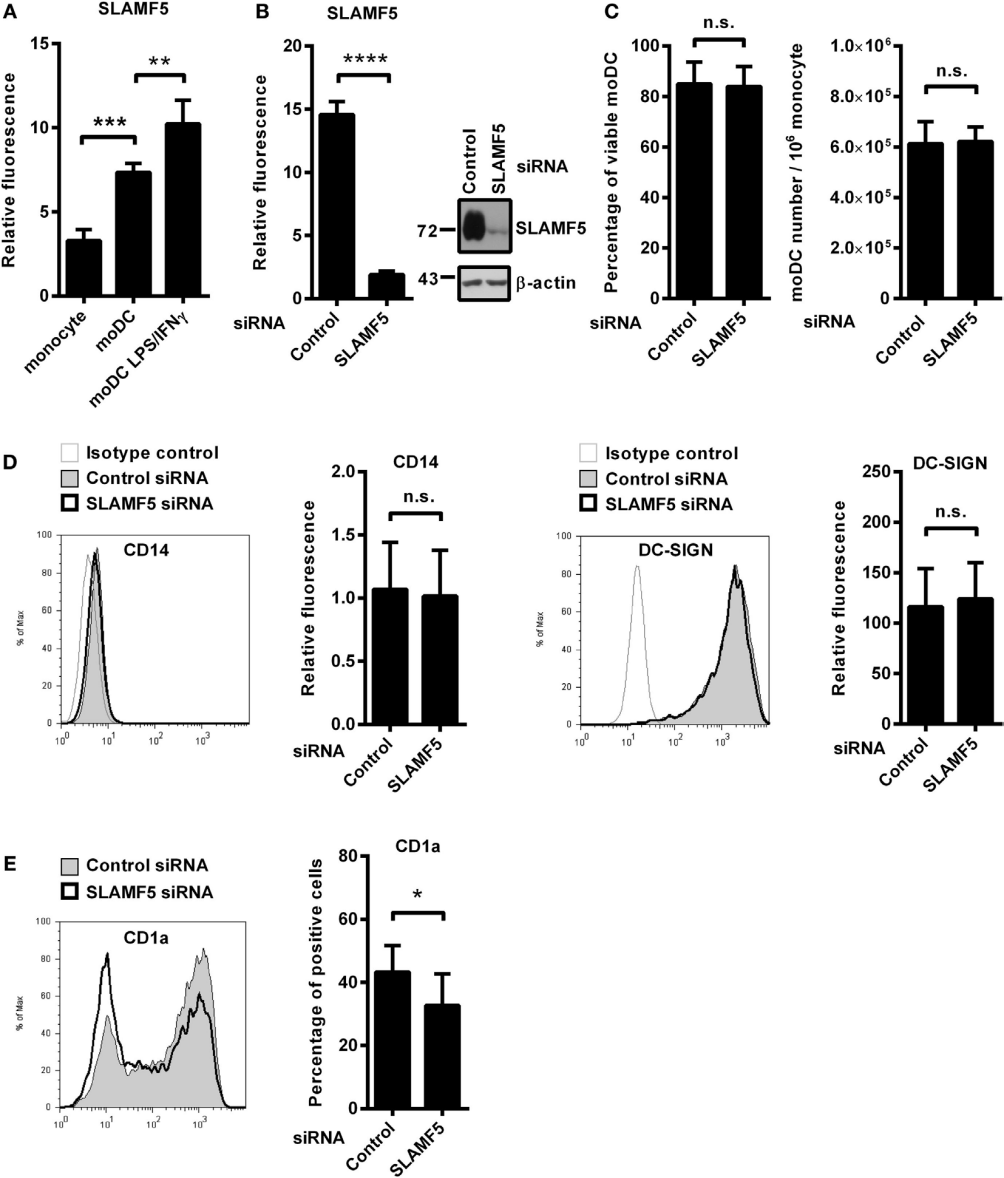
The effect of *SLAMF5* silencing on the phenotype of moDCs. **(A)** Cell surface expression of SLAMF5 on monocytes and on GM-CSF/IL-4-differentiated moDCs treated or not with LPS/IFNγ was assessed by flow cytometry. **(B)** Monocytes were transfected with the indicated siRNAs and differentiated into moDCs. On day 5, SLAMF5 expression was measured by flow cytometry and western blot analysis. β-actin was used as protein loading control. **(C)** Viability of control and *SLAMF5* knockdown moDCs was determined by 7-aminoactinomycin-D (7-AAD) dye exclusion. Bar graphs indicate percentage of cells negative for 7-AAD (left panel) and the total number of moDCs differentiated from 10^6^ monocytes (right panel). **(D)** Expression levels of CD14 and DC-SIGN in control and *SLAMF5* knockdown moDCs were measured by flow cytometry. Representative histograms show protein expression in control (thin line with gray shading) and knockdown cells (bolded black line) or staining with isotype control antibody (thin gray line). Bars show the relative fluorescence intensity values of CD14 and DC-SIGN, calculated using the respective isotype-matched control antibodies. The results shown are taken from four independent donors. **(E)** Representative histograms show CD1a expression in control (thin line with gray shading) and *SLAMF5*-silenced (bolded black line) moDCs. Bars show the percentage of CD1a^+^ cells. Data are presented as means ± SD of six independent experiments. Error bars indicate SD (*p < 0.05, **p < 0.01, ***p < 0.001, and ****p < 0.0001; ns, not significant).

In the process of *in vitro* differentiation of moDCs, expressions of several receptors with immune modulatory function are upregulated. We found that SLAMF5 is strongly induced when monocytes are differentiated into DCs by GM-CSF and IL-4 suggesting that SLAMF5 is required for the proper functions of immature DCs (Figure 1A). To evaluate the impact of SLAMF5–SLAMF5 interaction on moDC functions, we performed RNA interference targeting SLAMF5 on freshly isolated monocytes. Based on flow cytometry and western blot analyses, SLAMF5 expression was reduced by 80–95%, and silencing was maintained throughout the entire course of differentiation (Figure 1B). Although Binsky et al. have recently published that SLAMF5 acts as a survival receptor in chronic lymphocytic leukemia cells and its downregulation results in receptor induced cell death (31), under our experimental conditions neither the viability of moDCs nor the yield of differentiation was affected by *SLAMF5* silencing (Figure 1C). Next, we analyzed whether SLAMF5-deficient cells display any defects in moDC differentiation by measuring CD14, highly expressed on monocytes but downregulated on moDCs, as well as DC-SIGN (CD209), typically expressed by differentiated DCs. As shown in Figure 1D, cell surface expression of CD14 and DC-SIGN was identical in *SLAMF5*-silenced and control moDCs indicating that even grossly diminished levels of SLAMF5 did not affect the differentiation of monocytes into moDCs.

As simultaneous LPS and IFNγ stimuli further increased its expression level in moDCs (Figure 1A), we analyzed the potential role of SLAMF5 as a modulator of DC activation in response to LPS/IFNγ. Downregulation of *SLAMF5* expression nevertheless was not accompanied by severely impaired LPS/IFNγ-response as *SLAMF5*-silenced moDCs retained their capacity to enhance cell surface expression of markers associated with DC maturation in response to LPS/IFNγ stimulation. The amounts of HLA-A, B, C and HLA-DQ increased to the same extent after stimulation of control and *SLAMF5*-silenced cells. In addition, the expression of the co-stimulatory receptors CD40 and CD86 was identical in control and knockdown cells (Figure S1A in Supplementary Material). Interestingly, silencing of *SLAMF5* at an early phase of moDC differentiation led to a moderate, but significant decrease in the subset of CD1a^+^ DCs (Figure 1E). Further analysis of the above DC-maturation markers in the CD1a^+^ and CD1a^−^ DC-subsets, however, revealed no significant differences in response to *SLAMF5* silencing (Figures S1B–D in Supplementary Material).

### SLAMF5 Enhances Autophagy in moDCs

**Figure 2:**
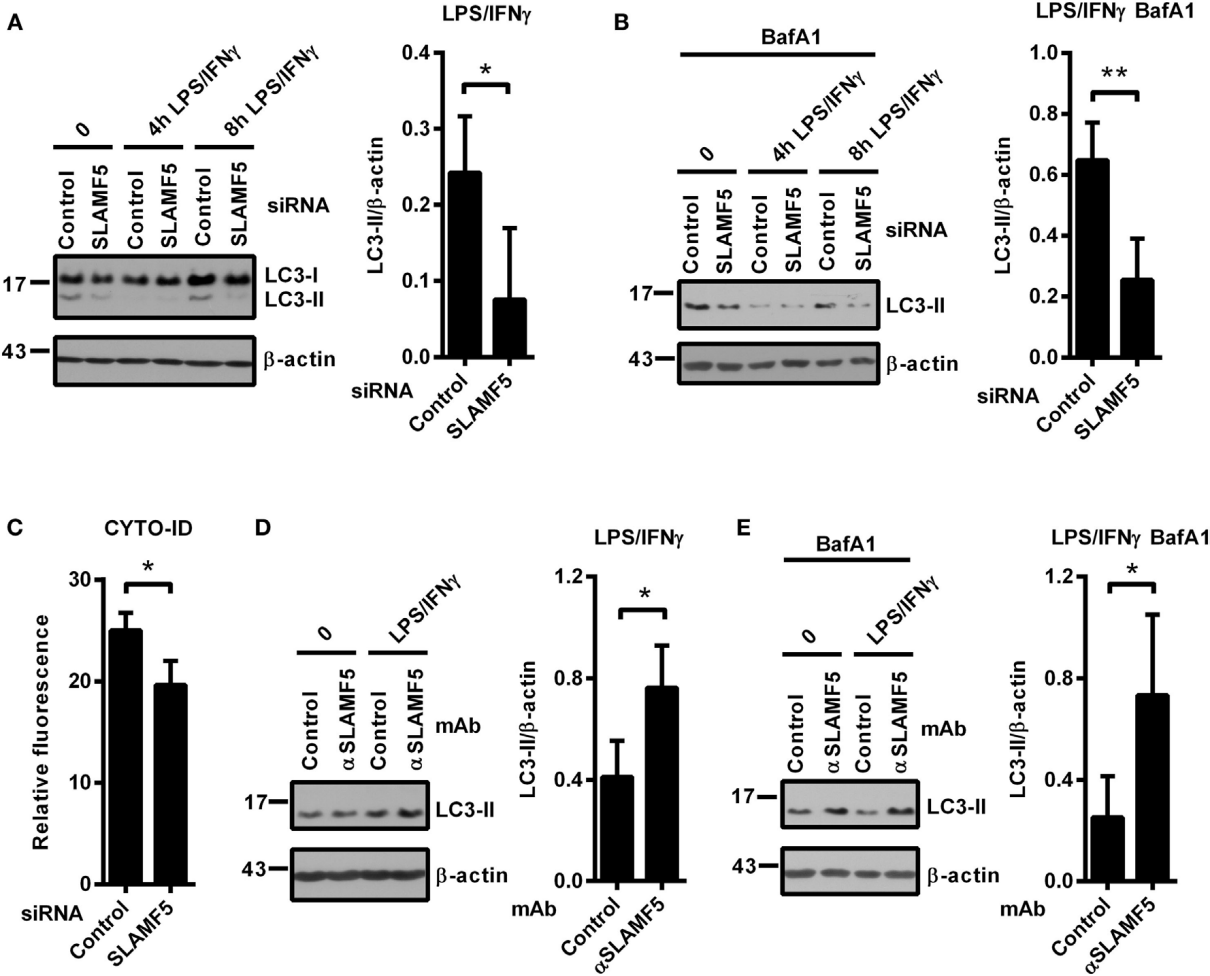
SLAMF5-mediated enhancement of dendritic cell autophagy under steady-state conditions and in response to LPS/IFNγ activation. Control and *SLAMF5*-silenced moDCs were stimulated or not with LPS/IFNγ for the indicated time periods in the absence [**(A)** cropped] or in the presence of 20 nM bafilomycin A1 (BafA1) applied for the last 2 h **(B)**. Conversion of LC3 was determined by western blotting. A representative immunoblot of four independent experiments is shown on the left; ratios of LC3-II and β-actin determined by densitometry are shown in the right panels. **(C)** Control and SLAMF5-depleted moDCs were stained with CYTO-ID. Fluorescence intensity was determined by flow cytometry. Graph depicts the relative fluorescence intensity of CYTO-ID obtained in four independent experiments **(D,E)**. moDCs were conditioned with 10 μg ml^−1^ SLAMF5-specific or control antibodies followed by cross-linking with 10 μg ml^−1^ F(ab′)_2_ fragment of goat anti-mouse IgG, then stimulated with LPS/IFNγ for 8 h in the absence **(D)** or in the presence **(E)** of 20 nM BafA1 applied for the last 2 h of the experiment. LC3 and β-actin levels were analyzed by western blotting; a representative blot and the mean ratios of LC3-II to β-actin from three independent experiments are shown. Data are expressed as the mean ± SD (*p < 0.05 and **p < 0.01).

In light of the recently established link between autophagy and SLAMF receptors (23, 25), we examined whether SLAMF5 partakes in the process of autophagy in resting or activated DCs. Microtubule associated protein 1 light chain 3 beta (LC3 hereafter) is widely used to monitor autophagy. During autophagy induction the processed LC3 is covalently coupled with phosphatidylethanolamine in the newly forming autophagosomal membrane (32). The cleaved cytosolic LC3 (called LC3-I) and the lipid-modified, autophagosome-associated LC3 (LC3-II) can be identified by their different electrophoretic mobility and the level of LC3-II correlates with the amount of autophagosomes (33). As displayed in Figure 2A, we detected lower LC3-II/β-actin ratios in *SLAMF5*-silenced moDCs than in control cells indicating the importance of SLAMF5 in maintenance of basal autophagy. To evaluate kinetic changes in autophagy during DC activation, we treated control and *SLAMF5* knockdown moDCs with LPS/IFNγ for 4 and 8 h. The expected transient, activation-dependent decrease in LC3-II levels (10, 12) was apparent 4 h past treatment with LPS/IFNγ both in control and *SLAMF5*-silenced moDCs. At 8 h past stimulation, however, LC3-II levels recovered in control but not in *SLAMF5*-silenced cells (Figure 2A; Figure S2A in Supplementary Material). Since LC3-II molecules localized in the inner membrane of autophagosomes are degraded during the autophagic process, lower levels of LC3-II may result from either decreased autophagosome formation or increased autophagic degradation (33). When BafA1, an inhibitor of autophagosome–lysosome fusion and lysosomal acidification (34) was applied LC3-II levels remained lower in the absence of SLAMF5 compared with controls (Figure 2B). As a control for the activity of BafA1, the extent of LC3-II accumulation is shown side by side in BafA1-treated and untreated samples (Figure S2B in Supplementary Material). This observation implies that rather than being involved in the maturation process of autophagosomes, SLAMF5 appears to be a positive regulator of autophagosome biogenesis. To further support SLAMF5-mediated regulation of autophagy we used CYTO-ID, a fluorescent dye that selectively labels autophagic vacuoles in live cells (35) and thus can be readily quantified by flow cytometry (36). In agreement with the results obtained by LC3 blotting, fluorescence intensity of CYTO-ID was significantly lower in *SLAMF5* knockdown cells than in controls (Figure 2C). Confocal microscopy analysis of CYTO-ID staining revealed punctate structures, the number of which increased in the presence of chloroquine (Figure S2C in Supplementary Material). Chloroquine inhibits maturation of autophagosomes by affecting lysosomal acidification, thereby resulting in accumulation of large CYTO-ID positive compartments (37). Therefore, the fluorescence signal detected by flow cytometry indeed resulted from accumulation of the dye in autophagic vacuoles.

If SLAMF5 is an important regulatory module of the autophagy pathway, increased signaling *via* SLAMF5 is expected to enhance autophagy. To test this, we used the SLAMF5-specific agonistic antibody 152.1D5 reported to induce SLAMF5 signaling both in T cells (38) and in chronic lymphocytic leukemia cells (31). Cross-linking of cell surface SLAMF5 with the 152.1D5 monoclonal antibody greatly increased LC3-II levels both in the presence and absence of BafA1 (Figures 2D,E). To exclude Fc receptor-mediated effects, an isotype-matched mAb was used as control in each experiment.

Collectively, these data demonstrate that SLAMF5 in moDCs potentiates basal autophagy and the recovery of autophagy after LPS/IFNγ activation.

### Autophagy Defect Observed in the Absence of SLAMF5 Is Not Mended by Rapamycin

**Figure 3:**
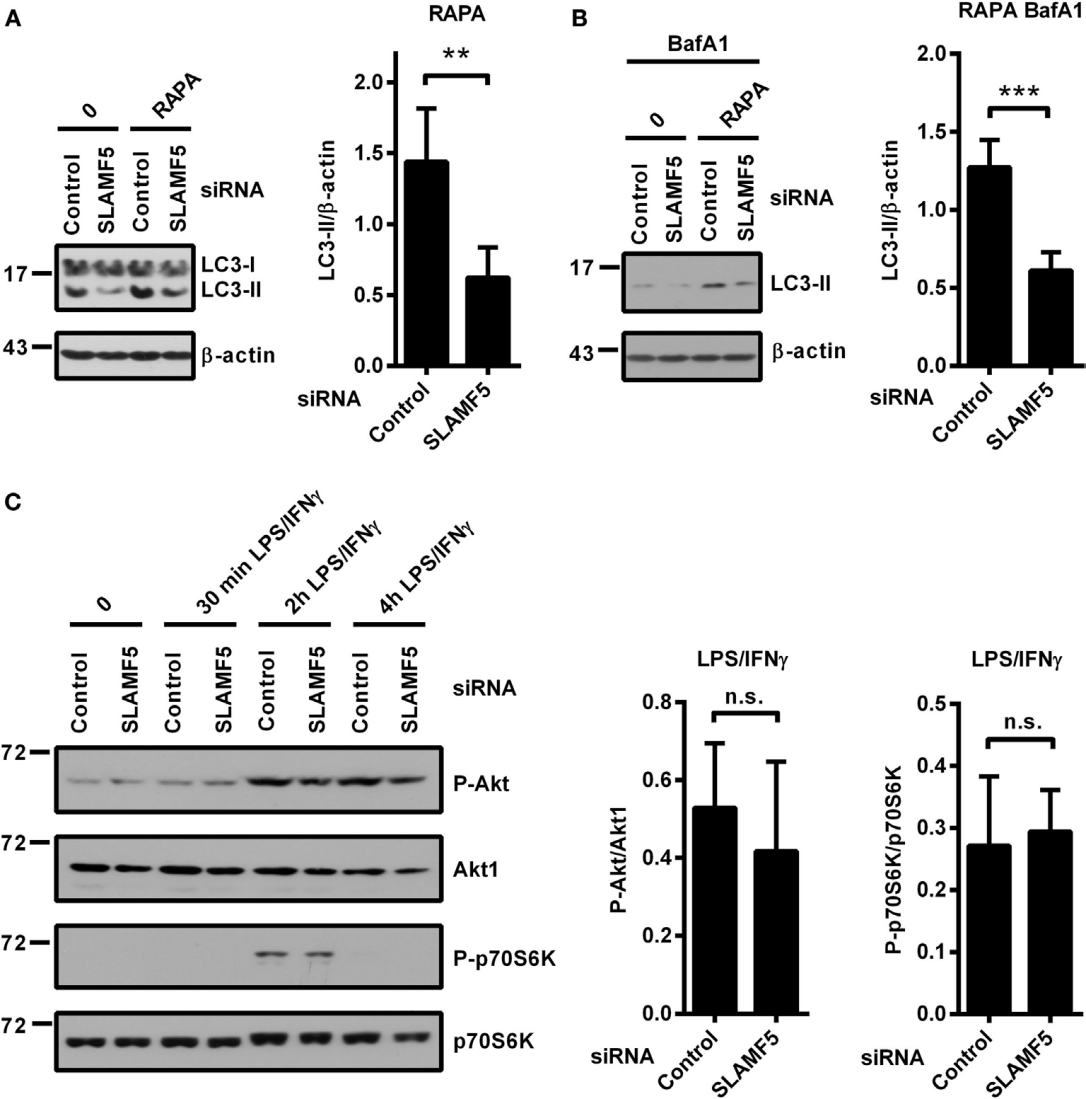
Enhancement of autophagy by SLAMF5 is not mediated by inhibition of the mTORC1 complex. **(A)** The autophagy-inducer rapamycin was applied to control or *SLAMF5*-silenced cells for 4 h. Autophagy was monitored by western blot analysis using an LC3-specific antibody. A representative blot of four independent experiments is shown on the left, and densitometry of LC3-II to β-actin ratios is depicted on the right panel. **(B)** Cells were treated as described in panel **(A)**, except that they were cultured in the presence of 20 nM bafilomycin A1 (BafA1) for the last 2 h. Data were analyzed as described in panel **(A)**. **(C)** *SLAMF5*-silenced or control monocyte-derived dendritic cells were activated with LPS/IFNγ, and phosphorylation of Akt and p70S6K was analyzed with immunoblotting. Representative blots of four independent experiments are shown. The phospho-proteins were normalized to the total levels of Akt1 and p70S6K. Data are expressed as mean ± SD (**p < 0.01 and ***p < 0.001; ns, not significant).

mTOR is one of the key negative regulators of autophagy (39). One potential mechanism by which SLAMF5 could enhance the level of autophagy was inhibition of the mTORC1 complex. To examine this scenario we used rapamycin (RAPA), a specific inhibitor of the mTORC1 complex and a well-known inducer of autophagy (39). Control and *SLAMF5*-silenced moDCs were treated with rapamycin for 4 h either in the presence or absence of BafA1. As shown in Figures 3A,B, the autophagy defect remained apparent in *SLAMF5*-silenced cells even when mTORC1 was suppressed, suggesting that SLAMF5-mediated regulation of autophagy is independent of mTOR activity. To further test this scenario, *SLAMF5*-silenced and control moDCs were activated with LPS/IFNγ, and phosphorylation of Akt, a kinase upstream of mTOR, and of the mTOR target protein p70S6K was monitored by western blot analysis. If SLAMF5 increases autophagy by inhibition of mTOR activity, silencing of *SLAMF5* should potentiate the Akt/mTOR/p70S6K pathway resulting in increased phosphorylation of these proteins. However, as shown in Figure 3C, the amounts of phospho-Akt and phospho-p70S6K were comparable in *SLAMF5*-silenced and control cells. The fact that rapamycin failed to restore the autophagy defect present in *SLAMF5*-silenced cells together with our findings that phosphorylation of Akt, and p70S6K was unaffected at diminished SLAMF5 protein levels strongly suggest that mTOR is not the main mediator of the autophagy pathway modulated by SLAMF5.

### SLAMF5 Regulates Autophagy *via* Preventing Proteasomal Degradation of IRF8

**Figure 4:**
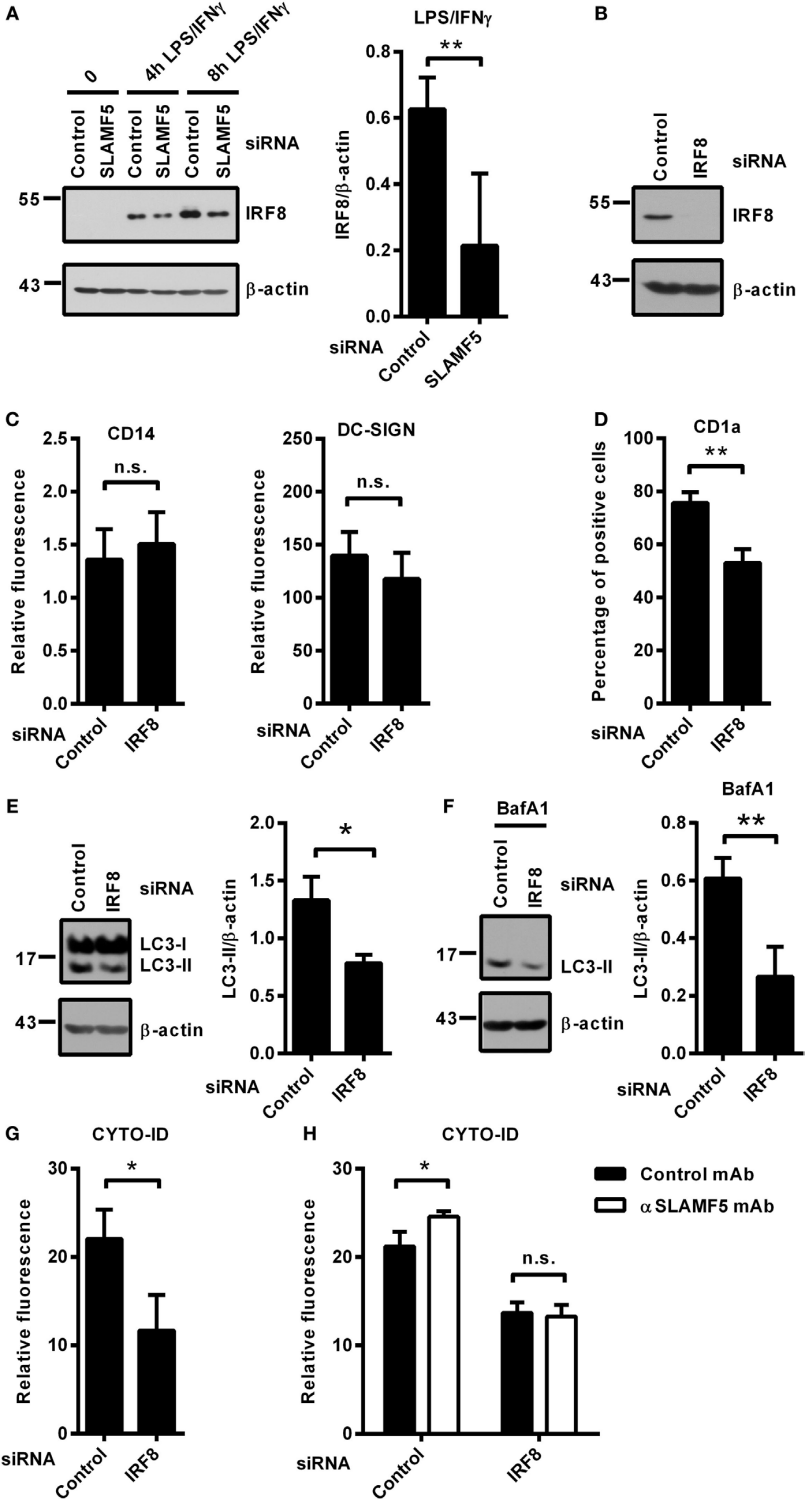
SLAMF5 controls the level of IRF8 protein, a positive regulator of autophagy in moDCs. **(A)** moDCs transfected with either control or an *SLAMF5*-specific siRNA were treated with LPS (100 ng ml^−1^) and IFNγ (10 ng ml^−1^) for the indicated time periods. IRF8 protein level was measured by immunoblotting, and the ratio of IRF8/β-actin was quantified from five independent experiments. **(B)** Efficiency of *IRF8* knockdown was tested on day 5 by western blot analysis; a representative blot is shown with β-actin as loading control. **(C)** Expression levels of CD14 and DC-SIGN as well as CD1a **(D)** were measured in control and *IRF8* knockdown moDCs by flow cytometry. Bars show the relative fluorescence intensity values or the percentage of positive cells. The results shown are taken from three independent experiments. LC3-II expression was measured in control or *IRF8*-silenced moDCs by immunoblotting in the absence **(E)** or in the presence of bafilomycin A1 (BafA1) **(F)**. One representative of three experiments is shown. Bar charts display the ratio of LC3-II/β-actin. **(G)** CYTO-ID staining of control and *IRF8*-silenced moDCs from three donors was analyzed by flow cytometry. **(H)** CYTO-ID staining of control and *IRF8*-silenced moDCs in which SLAMF5 was cross-linked with the SLAMF5-specific agonistic antibody 152.1D5 as described in Section “Materials and Methods.” Data are expressed as the mean ± SD (*p < 0.05 and **p < 0.01, ns, not significant).

Recently, Gupta and colleagues reported that IRF8 is expressed under steady-state conditions in murine bone marrow-derived DCs and its expression level is significantly increased in response to LPS/IFNγ treatment. Moreover, IRF8 was shown to switch on the expression of many genes involved in all phases of autophagy (13). Based on the central role of IRF8 in the process of autophagy, we hypothesized that the molecular mechanism by which SLAMF5 promotes autophagy may involve the regulation of IRF8 activity. To test this, first, we silenced *SLAMF5* and analyzed induction of IRF8 expression in response to treatment with LPS/IFNγ. As shown in Figure 4A, the amount of IRF8 protein in *SLAMF5*-silenced moDCs was significantly decreased. To gain more insight into the effects of decreased IRF8 levels on moDC differentiation and functions, *IRF8* expression was silenced using an *IRF8*-specific siRNA (Figure 4B). Interestingly, we found that silencing of *IRF8*, a lineage determining factor for myeloid cells, had no significant effect on the downregulation of CD14 expression or the induction of DC-SIGN expression associated with the differentiation process in the presence of IL-4 and GM-CSF (Figure 4C). Moreover, IRF8 expression was also not requisite for the expression of SLAMF5 that was significantly increased in *IRF8*-silenced cells (Figure S3 in Supplementary Material).

Importantly, the shift toward CD1a^−^ moDCs observed in *SLAMF5*-silenced cells was evident in *IRF8* knockdown cells (Figure 4D), consistent with a previous study by Granato et al., reporting that interference with the autophagic process in monocytes resulted in reduced expression of CD1a on moDCs (40). To test whether IRF8 acts as a regulator of autophagy in human moDCs, the intensity of autophagy was compared in *IRF8*-silenced and control moDCs. In line with its function in murine macrophages and DCs, and its suspected role as a mediator of SLAMF5’s effects, *IRF8*-silenced moDCs showed significantly decreased autophagy compared with controls. As shown in Figures 4E,F, the amount of LC3-II as well as the fluorescence intensity of CYTO-ID were significantly decreased (Figure 4G). These findings demonstrate that similar to murine bone marrow-derived DCs, IRF8 is used for the regulation of autophagy in human moDCs. To determine whether SLAMF5 and IRF8 are part of the same or different autophagy regulatory pathways, SLAMF5 was cross-linked with the above described 152.1D5 mAb antibody on moDCs transfected with control or *IRF8*-specific siRNA. As shown in Figure 4H, cross-linking of SLAMF5 significantly increased autophagy in moDCs transfected with the control oligonucleotides; however, this induction was dependent on the presence of IRF8. This observation established IRF8 as part, and a downstream element of the SLAMF5 autophagy regulatory pathway.

**Figure 5:**
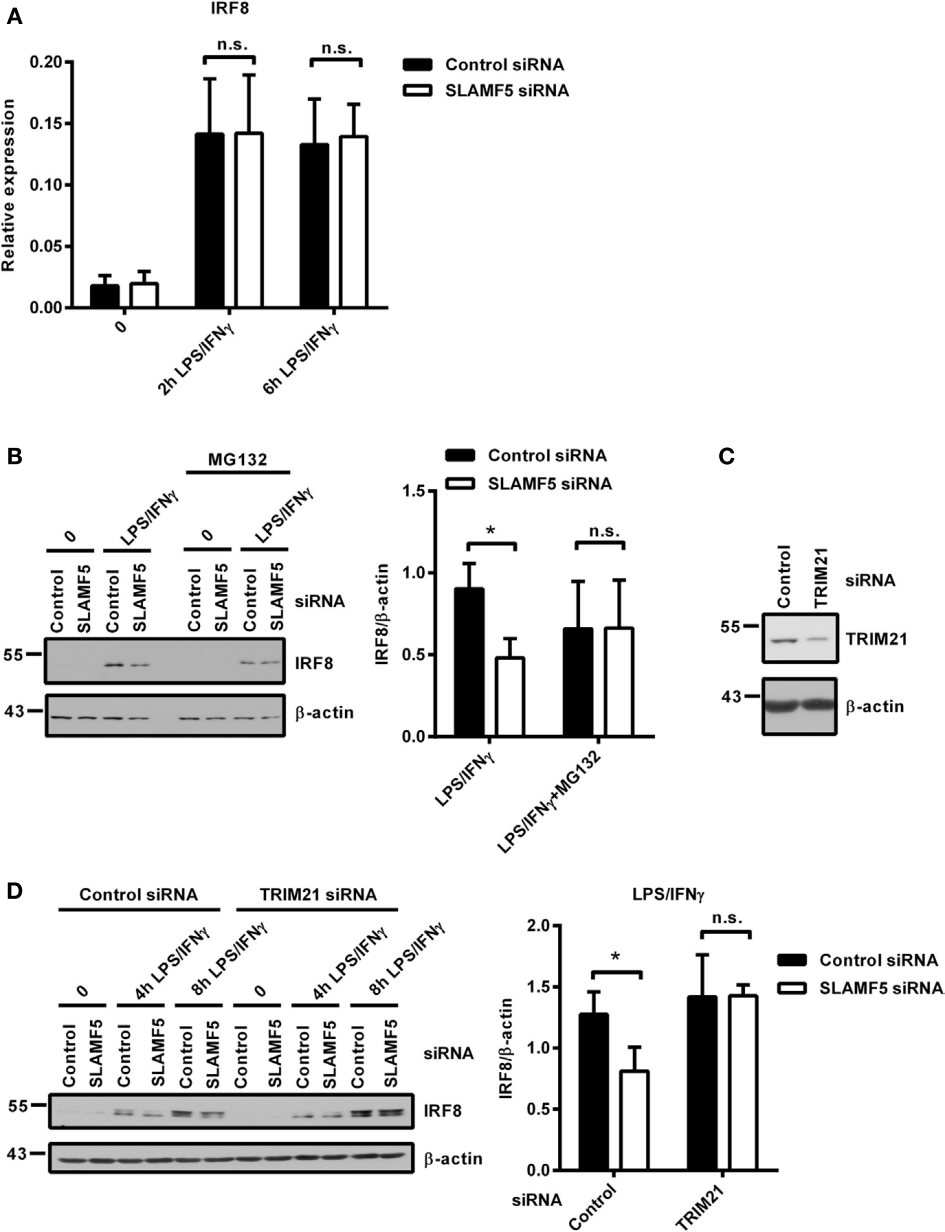
Proteasomal degradation of IRF8 is dependent on the E3-ubiquitin ligase TRIM21. **(A)** Kinetics of *IRF8* mRNA expression were analyzed by quantitative PCR in five donors after treatment of control and *SLAMF5* knockdown moDCs with LPS/IFNγ for the indicated time periods. **(B)** Control and *SLAMF5* knockdown moDCs were stimulated with LPS/IFNγ for 8 h and, where indicated, 1 µM MG132 was added 2 h before harvesting. A representative immunoblot (left) and densitometric quantification (right) of three immunoblots from independent experiments are shown. **(C)** Efficiency of *TRIM21* knockdown was tested on day 5 by western blot analysis; a representative blot is shown with β-actin as loading control. **(D)** *SLAMF5* expression alone or together with *TRIM21* expression was silenced by transfection of moDCs with the indicated set of gene-specific or control siRNAs. Transfected cells were activated with LPS/IFNγ for the indicated time periods. IRF8 protein levels were determined by western blotting. One representative blot (left) and densitometry of three independent experiments (right) are shown. Data are expressed as the mean ± SD (*p < 0.05; ns, not significant).

Protein levels are determined by the rate of transcription followed by translation as well as the rate of protein degradation. When *SLAMF5*-silenced and control moDCs were activated by LPS/IFNγ, both the kinetics and the intensity of *IRF8*-transcription were found to be identical between silenced cells and controls (Figure 5A) indicating the lack of SLAMF5-mediated regulation at the level of *IRF8*-transcription. Given that IRF8 activity and its stability are regulated by polyubiquitination and subsequent proteasomal degradation (15, 17), we examined whether degradation of IRF8 by the proteasome was affected by SLAMF5. When moDCs were activated by LPS/IFNγ in the presence of the proteasome inhibitor MG132, IRF8 levels in *SLAMF5* knockdown moDCs were completely restored (Figure 5B). As a control, enrichment of ubiquitin-linked proteins in the presence of MG132 was confirmed by probing identical blots with a ubiquitin-specific antibody (Figure S4 in Supplementary Material). Since ubiquitination of IRF8 by the E3-ubiquitin ligases TRIM21 (Ro52) (15) and casitas B-lineage lymphoma (c-Cbl) (17) has been described in murine macrophages, we tested whether IRF8 degradation in *SLAMF5*-silenced cells required the participation of these ligases. To this end, first, we confirmed induction of TRIM21 in response to LPS/IFNγ (Figure S5 in Supplementary Material) and its silencing in the presence of *TRIM21* gene-specific siRNA (Figure 5C). Next, *SLAMF5* alone, or in combination with *TRIM21*, was silenced, and the level of IRF8 protein was measured in moDCs treated with LPS/IFNγ. As shown in Figure 5D, IRF8 degradation in *SLAMF5*-silenced moDCs was blocked when cells were co-transfected with the *TRIM21*-specific, but not with the control oligo. This finding clearly showed that increased degradation of IRF8 in the absence of SLAMF5 was dependent on the presence of TRIM21. In similar experiments, IRF8 was not rescued from degradation by silencing the E3-ubiquitin ligase *c-Cbl* (data not shown).

Two single SH2 domain-containing proteins, the SLAM-associated protein and the Ewing sarcoma activated transcript-2 (EAT-2) are major transducers of SLAMF-receptor signaling (19, 41, 42). Based on its reported expression in antigen-presenting cells (43) and a recent report describing it as an enhancer of the autophagy process (23), EAT-2 emerges as a potential adapter transmitting SLAMF5 signals. However, when EAT-2 expression was thoroughly examined in our model, we found EAT-2 expressed in human monocytes, but abruptly downregulated in response to the used DC differentiation signals. The EAT-2 protein was absent both in resting and LPS/IFNγ-activated moDCs (Figure S6 in Supplementary Material), indicating that SLAMF5 drives autophagy in moDCs in an EAT-2-independent manner.

Taken together, unlike its induction of gene expression and translation, the stability of the IRF8 protein is strongly affected by the presence of SLAMF5 that appears to control the activity of TRIM21. These data also support a mechanism connecting SLAMF5 to autophagy as part of the regulatory cascade sustaining activity of IRF8, a major transcription factor of autophagy-related genes.

### *SLAMF5* and *IRF8* Silencing in Monocytes Results in Development of moDCs with Overlapping Changes in Cytokine Secretion

**Figure 6:**
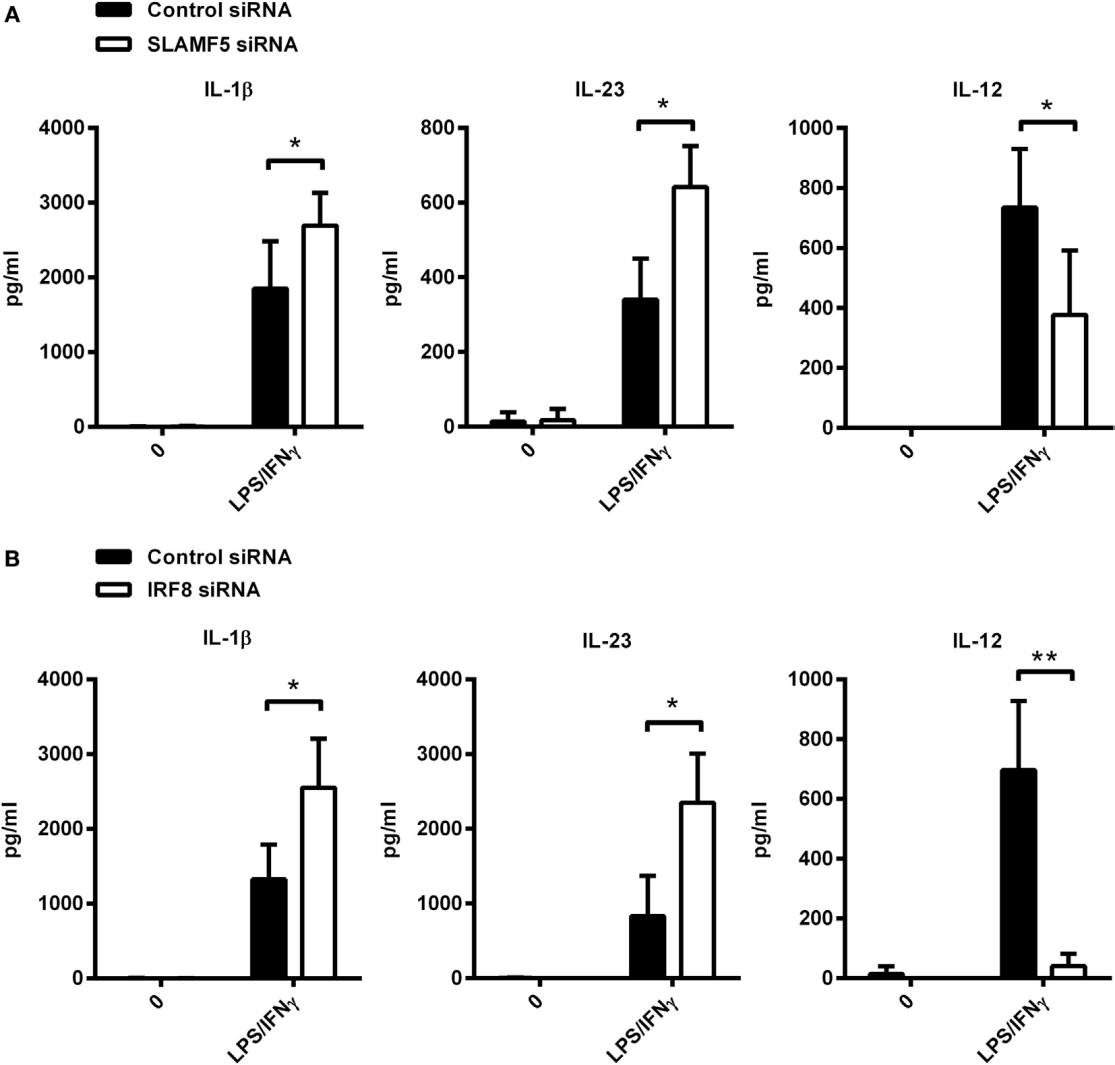
Cytokine production of *SLAMF5*- and *IRF8*-silenced moDCs. *SLAMF5*- **(A)** or *IRF8*-silenced **(B)** moDCs were left untreated or stimulated with LPS/IFNγ, and their cytokine productions were compared with cells transfected with control oligonucleotides. Cytokine concentrations in supernatants were determined at 8 h (IL-1β and IL-23) or 12 h (IL-12) by ELISA. Lack of bars indicates cytokine secretion below the limit of sensitivity for the ELISA. Data are presented as means ± SD of four independent experiments (*p < 0.05 and **p < 0.01).

To further support our hypothesis that the impact of *SLAMF5* silencing on DC functions is the consequence of IRF8 degradation, hereinafter, we examined whether depletion of IRF8 could induce similar functional changes to those seen in *SLAMF5*-silenced moDCs. Given the well-characterized role of autophagy in the inflammatory response, the contribution of SLAMF5 and IRF8 to cytokine production of moDCs was examined. To this end, we measured the amount of pro-inflammatory cytokines in the supernatant of control and knockdown moDCs exposed to LPS/IFNγ. *SLAMF5*- (Figure 6A) or *IRF8*-silenced cells (Figure 6B) released significantly higher amounts of IL-1β and IL-23 than did control cells, implying that both SLAMF5 and IRF8 may function to restrain excessive inflammatory reactions. On the contrary, *SLAMF5*-silenced or *IRF8* knockdown cells produced less IL-12 than control cells. Remarkably, these experiments also showed that IL-12 production in response to LPS/IFNγ stimulation was fully IRF8 dependent (Figure 6B). The finding that loss of IRF8 replicated several changes in DC phenotype (Figures 1D,E and 4C,D) and cytokine production observed in *SLAMF5*-silenced cells, lends further support to a model in which SLAMF5 and IRF8 are part of the same regulatory pathway.

**Figure 7:**
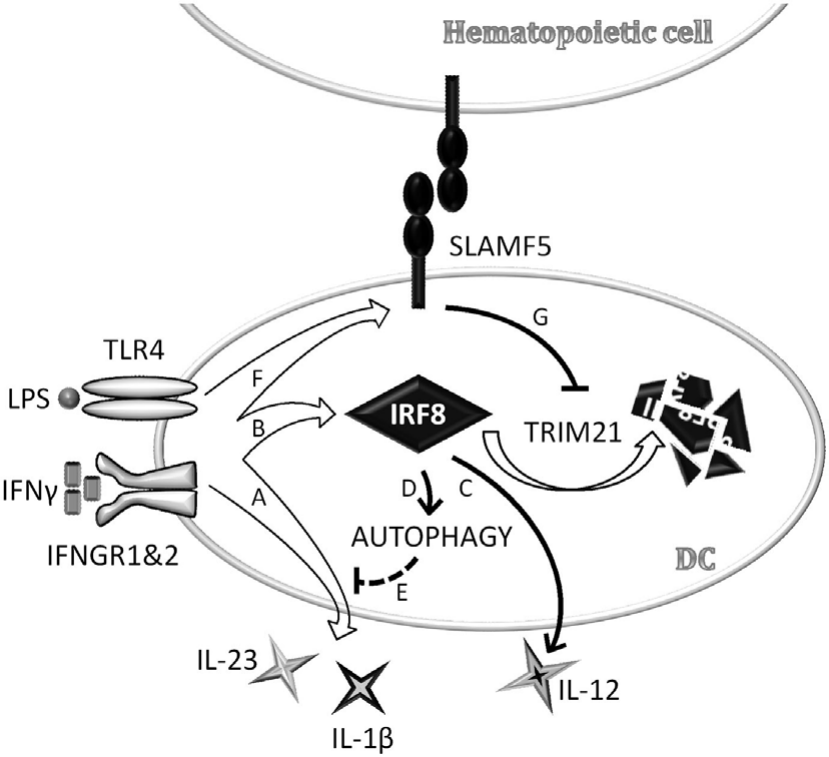
Schematic model depicting signaling lymphocyte activation molecule family (SLAMF) 5-mediated modulation of autophagy and cytokine production in monocyte-derived dendritic cells (moDCs) *via* stabilization of interferon regulatory factor 8 (IRF8). In an ongoing infection with Gram-negative bacteria, dendritic cells (DCs) are exposed to LPS and IFNγ that are known to reduce autophagy, presumably to prevent exposure of autoreactive T cells to self-antigens in the presence of strong co-stimulatory signals. In the early phase of DC activation, concomitant with the secretion of pro-inflammatory cytokines **(A)**, IRF8 expression is induced **(B)**. IRF8 in turn promotes the production of IL-12 **(C)** and restores autophagy **(D)** to control overproduction of potentially self-destructive cytokines, such as IL-1β and IL-23 **(E)**. We found that SLAMF5 is upregulated in response to LPS/IFNγ **(F)** and inhibits TRIM21-mediated proteasomal degradation of IRF8 **(G)**. By this mechanism, SLAMF5 acts as a positive regulator of autophagy in moDC, likely impacting on the outcome of antimicrobial responses as well as on autophagy-driven pathologies.

Taken together, data presented above identifies SLAMF5 as a novel cell surface-expressed regulator of DC autophagy operating *via* stabilization of one of the autophagy master-regulator transcription factors, IRF8 (Figure 7).

## Discussion

SLAMF receptors regulate various stages of the immune response including the differentiation of innate and classical T cell subsets as well as the T- and B cell memory response (19, 21, 41). Consequently, in addition to antimicrobial responses, SLAMF receptors have been shown to contribute to pathological autoimmune responses, i.e., colitis and systemic lupus erythematosus (44–46), as well as to neuropsychiatric diseases with inflammatory and neurodegenerative etiologies (47, 48). Despite its ubiquitous expression in practically all hematopoietic lineages, mice carrying defective *SLAMF5* alleles at both chromosomes have a relatively mild phenotype. The main defect appears to be restricted to germinal center formation, specifically due to lack of proper adhesion and functional interaction between T follicular helper (Tfh) and B cells, but not DCs (28). However, normal DC functions in the absence of SLAMF5 may be explained by compensatory mechanisms implemented by other SLAMF members. Alternatively, appearance of the unique functions of SLAMF5 may require specific environmental challenges, yet to be identified. In support of this latter scenario, SLAMF5 has been shown to regulate TCR-, BCR-, and FCεRI-mediated signal-transduction (26, 28, 49). The function of SLAMF5 in myeloid cells, however, remains poorly understood. In a single report, *SLAMF5* knockdown was found to decrease LPS-induced TNF-alpha and IL-6 production of murine bone marrow-derived macrophages (50). In our work, we set up experiments to analyze the function of SLAMF5 in human moDCs. When SLAMF5 expression was decreased by 80–95% using transfection of specific siRNA, human peripheral blood monocytes readily differentiated into moDCs, suggesting that SLAMF5 may not be required for the main steps of moDC differentiation. In addition, although SLAMF5 was shown to act as a survival factor in certain tumor cell lines (31), viability of SLAMF5-deficient moDCs appeared to be identical to that of control cells, suggesting that the survival-promoting effect of SLAMF5 may be context dependent, lineage-specific, or even restricted to transformed cells.

Nevertheless, production of IL-1β and IL-23 was significantly enhanced by cells with reduced SLAMF5 levels while IL-12 secretion was diminished compared with controls. These functional changes occurred in the absence of altered expression of receptors associated with DC activation including HLA proteins or the co-stimulatory molecules CD40 and CD86. Thus, SLAMF5-mediated modulation of cytokine production without affecting co-stimulatory receptor expression may allow DCs to alter polarization of naïve T cells while supporting their proliferation. In this regard, independent regulation of DC maturation and inflammatory cytokine production has been described by the D’Oro group in TLR ligand-activated DCs treated with the pan-Src-kinase inhibitor PP1 or PP2 (51, 52).

More rigorous analysis of the SLAMF5-deficient moDCs identified a consistent, albeit moderate decrease of CD1a expression that together with an increase in IL-1β and IL-23 production was reminiscent of the phenotype of DCs treated with autophagy inhibitors (40). Inhibition of autophagy, either with the PI3K inhibitor 3-methyladenine or with siRNA against the autophagy protein Atg7 significantly increased IL-1β production of human PBMC stimulated with either LPS (53) or *Mycobacterium tuberculosis* (Mtb) (54). Likewise, LPS-activated human moDCs produced more IL-23 under such circumstances (55). It has also been reported that IL-1β, produced following autophagy inhibition in DCs, acted in an autocrine manner to drive IL-23 secretion and this appears to be the mechanism through which autophagy exerts its effects on IL-23 (55). In our experiments, in response to LPS/IFNγ, *SLAMF5*-silenced moDCs showed a similar increase of IL-1β and IL-23 production as autophagy-deficient cells, indicating that SLAMF5 may act as a positive regulator of autophagy in moDCs. Our hypothesis that SLAMF5 is a novel cell surface regulator of autophagy was confirmed by two complementary methods; by monitoring conversion of LC3 into its lipid-modified form, and monitoring accumulation of CYTO-ID, an autophagosome-specific dye. In our experiments, however, impaired autophagy was strikingly accompanied by decreased IL-12 secretion, which seems to contradict an earlier study reporting that mice lacking the autophagy protein Atg5 in myeloid cells secrete higher amounts of IL-12 in response to infection with Mtb (56). This discrepancy, however, may be readily resolved by the IRF8 dependence of LPS/IFNγ-induced IL-12 production in macrophages (14) and, as our results demonstrate, in human moDCs. In addition to IL-12, the transcription of multiple components of the autophagy machinery was recently shown to be under the control of IRF8 in murine DCs. Our work shows that, similarly to murine cells, IRF8 has a pivotal role in the autophagic process of human moDCs. Based on these findings we propose that DC autophagy is controlled by SLAMF5 *via* an IRF8-dependent mechanism. In support of this, we presented evidence that cross-linking with a SLAMF5-specific agonistic antibody significantly increased the autophagic flux of human moDCs both under steady-state conditions and following activation with LPS/IFNγ. Importantly, this effect was reversed by silencing *IRF8* expression of moDCs. Several studies have demonstrated the importance of posttranslational modifications in the regulation of IRF8 activity (15, 17). Pretreatment with the proteasome inhibitor MG132 restored IRF8 protein levels of *SLAMF5*-silenced cells indicating that in moDCs IRF8 is at least in part controlled by SLAMF5 *via* modulating its degradation by the proteasome.

Taken together, data presented in this work strongly suggest that SLAMF5 controls the production of key inflammatory cytokines *via* regulating autophagy, mediated at least in part by fine-tuning proteasomal degradation of one of the autophagy master regulators, IRF8. Regarding the molecular machinery involved in the described SLAMF5-dependent increase of IRF8 stability, we identified the E3-ubiquitin ligase TRIM21 as a mediator of IRF8 degradation in *SLAMF5*-silenced moDCs.

However compelling the mechanism of IRF8 control by the SLAMF5 signaling pathway in the regulation of autophagy and inflammatory cytokine production regulation seems, we cannot exclude contribution of other pathways. For example, similar to the mechanism described for SLAMF1 and SLAMF4, SLAMF5 may partake in the formation of the Beclin-1/Vps34 autophagy-associated regulatory complex (22–24). Although much of the molecular details of autophagy regulation are yet to be clarified, SLAMF5 should influence the early events of autophagy, steps independent of autophagosome maturation as well as mTOR, as neither BafA1 nor rapamycin rescued the defect seen in *SLAMF5*-silenced moDCs.

Pharmacological modulation of the autophagy process is of great significance (57). The close to ubiquitous expression of SLAMF5 in hematopoietic cells provides an opportunity to selectively augment autophagic functions in these cells. Immune cell-specific autophagy regulators on one hand could decrease the unwanted pleiotropic effects associated with manipulation of systemic autophagy, and at the same time, increase cell-extrinsic effects of autophagy that operate in the context of host defense.

MoDCs have been reported to be present in the mammalian brain during infections controlling reactive T-lymphocyte influx and contributing to neurodegenerative processes (58). Furthermore, moDCs are also thought to be crucial in brain immunosurveillance, and key players in various neuropathologies associated with Alzheimer’s disease (AD) and Parkinson’s disease (59). In AD, impaired clearance of amyloid-β plaques and tau protein tangles was shown in an SLAMF receptor and Beclin-1 interactome-dependent manner reflecting on the critical involvement of defective autophagy and phagocytosis in AD pathogenesis (48).

Based on the central role of autophagy in antimicrobial immunity and inflammation, autophagy upregulation in immune cells can be beneficial in infectious diseases (56). In this regard, Garcia et al. have recently reported that autophagy was induced by Mtb-derived antigens in the adherent population of PBMC of Mtb carriers. The level of autophagy was IFNγ dependent and showed a positive correlation with the level of IFNγ produced by the patient’s PBMC (60). Moreover, the same group has shown earlier that T cells isolated from patients with favorable clinical response to Mtb (high responders) had increased expression of SLAMF1 compared with those derived from low responders (61, 62). Interestingly, they also demonstrated that IL-17 and SLAMF1 have opposing effects on IFNγ production in tuberculosis patients (62). These findings are in line with our earlier results in LPS-activated moDCs showing a positive effect of SLAMF1 on the IL-12–IFNγ axis in the context of Th1-cell differentiation (63). In this article, we report that SLAMF5 modifies the cytokine profile of LPS/IFNγ-stimulated moDCs in a manner that would support Th1 cell differentiation in expense to Th17 cell differentiation. However, this interpretation may be overly simplistic. As described by Schmitt et al., IL-12 controls the differentiation of IL-21-producing Tfh-like cells, and induces IL-21 secretion from memory CD4^+^ T cells (64). These findings, together with the well documented role of SLAMF5 in the differentiation of murine Tfh cells imply that SLAMF5 in human DCs may also promote commitment of naïve CD4^+^ T cells toward the Tfh lineage. Altogether, our findings suggest that in human DCs, SLAMF5 supports intracellular pathogen-induced immune responses by both increasing the level of autophagy and by modulation of T-cell differentiation. Further research is required to understand the complexity of the underlying mechanisms, and to establish, to what extent, modulation of autophagy using agonistic SLAMF5-specific antibodies may be used to improve current therapies for various human diseases.

## Author Contributions

AL and ZA designed the study. ZA, DB, KP, AS, and AL performed experiments. Statistical analysis was done by ZA; confocal microscopy by GV. TB, AB, ER, and PE contributed with essential reagents and made significant conceptual contributions to the project. ZA, PE, and AL wrote the article. All the authors reviewed and approved the final version of the manuscript.

## Conflict of Interest Statement

The authors declare that the research was conducted in the absence of any commercial or financial relationships that could be construed as a potential conflict of interest. The reviewer MG and handling editor declared their shared affiliation.

## Supplementary Material

Figure S1 Phenotypic maturation of *SLAMF5*-silenced moDCs. **(A)** moDCs were exposed to 100 ng ml^−1^ LPS and 10 ng ml^−1^ IFNγ for 24 h. Cell surface expression of HLA-A, B, C, HLA-DQ, CD40, and CD86 was measured by flow cytometry; values are expressed as reléative fluorescence. Expression of the above cell surface receptors was measured on the CD1a^+^ **(B)** and CD1a^−^ **(C)** subsets of control and *SLAMF5*-silenced moDCs. Bars show the relative fluorescence intensity values **(D)** Data are presented as means ± SD of three independent experiments. (**p* < 0.05 and ****p* < 0.001; ns, not significant).

Figure S2 Additional information to SLAMF5-mediated enhancement of dendritic cell autophagy. **(A)** This panel shows the uncropped version of Figure 2A. **(B)** Analysis of basal level of autophagy in control and *SLAMF5*-silenced cells. Control and *SLAMF5*-silenced moDCs were left untreated or incubated with 20 nM bafilomycin A1 (BafA1) for 2 h. LC3 and β-actin levels were analyzed by western blotting. In this panel, samples of BafA1-treated and untreated cells are run on the same gel. Please note the robust accumulation of LC3-II in the BafA1-treated samples! BafA1-treated samples are badly overexposed precluding quantitation of LC3-II readouts of BafA1-treated and untreated samples from the same gel. Thus, samples derived from BafA1-treated and untreated samples were run and analyzed separately. Of note, at shorter exposure times, LC3-I band is not visible on blots of the BafA1-treated samples. **(C)** Autophagosome formation was visualized by CYTO-ID staining followed by confocal microscopy in control and *SLAMF5*-silenced moDCs in the presence or absence of 50 μM Chloroquine, an inhibitor of lysosomal acidification. Representative images show appearance of fluorescent puncta due to vacuolar localization of CYTO-ID in autophagosomes. Nuclei are highlighted by Hoechst 33342 staining. FOV: 57.4 μm × 44.9 μm.

Figure S3 SLAMF5 expression in *IRF8* knockdown moDCs. On day 5 of moDC differentiation, cell surface expression of SLAMF5 in *IRF8*-silenced or control moDCs was measured by flow cytometry. Values are expressed as relative fluorescence and are the mean ± SD of five independent experiments (**p* < 0.05).

Figure S4 Accumulation of ubiquitinated proteins in the presence of the proteasome inhibitor MG132. This panel shows a representative blot of control experiments to confirm the activity of MG132 in our experimental system. MoDCs were treated as described in the legend for Figure 5B. Western blot was developed by a ubiquitin-specific antibody as described in Section “Materials and Methods.”

Figure S5 TRIM21 expression is enhanced in response to treatment with LPS/IFNγ. *SLAMF5* knockdown or control moDCs were exposed to 100 ng ml^−1^ LPS and 10 ng ml^−1^ IFNγ for 8 h. TRIM21 protein levels were determined by western blot analysis. A representative of three independent experiments is shown. Bar chart on the right displays TRIM21 protein levels of *SLAMF5*-silenced or control moDCs relative to β-actin (**p* < 0.05).

Figure S6 EAT-2 expression levels in human monocytes and moDCs. Western blot analysis of EAT-2 expression in monocytes and moDCs on day 5 of *in vitro* differentiation treated or not with LPS/IFNγ. A representative blot of three independent experiments is shown, with β-actin as loading control.
